# Use of Bosentan in neonatal post cardiac surgery pulmonary hypertension

**DOI:** 10.4103/0974-2069.58325

**Published:** 2009

**Authors:** Ravindra Pawar, Pankaj Kasar, Swati Garekar, Snehal Kulkarni

**Affiliations:** Department of Pediatric Cardiology, Pediatric and Congenital Heart Center, Wockhardt Hospital, Mumbai, India

**Keywords:** Bosentan, neonatal pulmonary hypertension, total anomalous pulmonary venous connection

## Abstract

We report the use of Bosentan in the post-operative period of a neonate with obstructed infradiaphragmatic total anomalous pulmonary venous connection and severe pulmonary arterial hypertension. To our knowledge, this is the first report of use of Bosentan in this situation.

## CASE REPORT

A 1-month-old female presented to us with cyanosis and feeding difficulty since birth. Her oxygen saturation was 30%. An echocardiography revealed an infradiaphragmatic total anomalous pulmonary venous connection (TAPVC )with obstruction at the hepatic vein level (mean gradient of 16 mmHg). There was a moderate sized patent ductus arteriosus (PDA) with a right to left shunt across it, and a moderate sized atrial septal defect (ASD) with right to left shunt.

She underwent rerouting of TAPVC with closure of ASD and PDA on the same day. The pulmonary artery (PA) line was in place for post operative monitoring of the PA pressure. In the post-operative intensive care unit, she was kept sedated and paralyzed, mildly hyperventilated on high inspired oxygen, and received ionotropic supports including milronone. A post-operative echocardiography revealed an unobstructed flow from the pulmonary venous chamber into the left atrium. PA pressure was persistently near systemic levels with frequent pulmonary arterial hypertension (PAH) crises on minimal stimulation. On postoperative day (POD)1, inhaled nitric oxide (iNO) was added and had to be increased to 40 ppm. Oral sildenafil (5 mg/kg/day) was started simultaneously. The patient persisted to have PAH crises with resultant systemic hypotension and desaturation.

**Figure 1:**
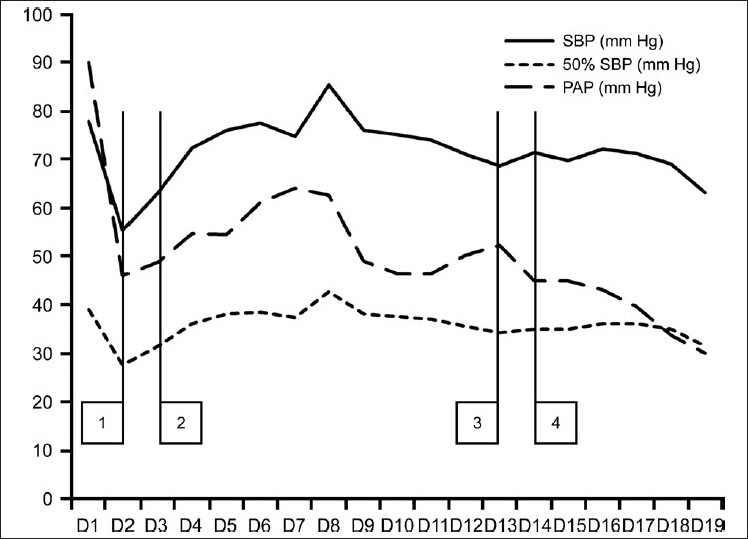
Comparison of the mean daily systolic blood pressures and mean daily pulmonary artery systolic pressures from admission to POD19. Vertical bar (1) represents surgery; (2) day of starting inhaled nitric oxide and Sildenafil; (3) starting Bosentan; (4) stopping inhaled nitric oxide

Extra corporeal membrane oxygenator (ECMO) facility is not available at our centre and the patient could not be weaned off the ventilator. Bosentan was started at 15.625 mg (1/8^th^ of 125 mg tablet) once daily on POD 11. There was a dramatic decrease in PA pressure [Figure 1] after starting Bosentan and iNO could be tapered off and omitted safely 24 hours after starting bosentan without a rebound increase in PA pressure. PA pressure stabilized to less than 50% systemic pressure and there were no further episodes of PAH crisis. The patient was weaned off the ventilator and extubated on POD 18. She was discharged on day 28 with Sildenafil and Bosentan. Before being discharged, an echocardiography revealed trivial tricuspid regurgitation with PA systolic pressure of 35 mmHg.

## DISCUSSION

Infants with obstructed TAPVC have post-capillary PAH. Over time, pulmonary arteries develop medial hypertrophy and intimal thickening.[1] After surgical relief of the obstruction, it may take weeks or months for the PAH to resolve. Due to limited reserves and additional cardio-pulmonary bypass related injury, the neonatal heart may not tolerate the PAH immediately after the operation. This manifests as acute right ventricular failure and low cardiac output creating a hemodynamically unstable patient.[2]

Post-operative management of neonates with PAH includes oxygen, alkalosis, sedation, paralysis, and inotropic support. Other therapies that are available are inhaled NO, prostacyclin therapy, phosphodiesterase inhibitors, and endothelin receptor antagonists.

Our patient was being treated with maximum strengths of iNO and phosphodiesterase inhibitors and persisted to have PAH and PAH crises until the addition of Bosentan.

Bosentan is an endothelin receptor antagonist. Endothelin is a potent vasoconstrictor peptide that is present in the vascular endothelial cell. It has two types of receptors, A and B, of which Type A mediates vasoconstriction. Bosentan is an antagonist to both receptors. It has been shown to reduce pulmonary vascular resistance and pulmonary arterial hypertension.[3] However, there are no randomised controlled trials using Bosentan in newborns or infants. In a retrospective study of 48 children (9 months or older) with congenital heart defects with PAH (24 unrepaired), Bosentan was given at a target dose of 31.25 mg/day.[4] There were only 3 infants below 10 kg. One-half of the target dose (15.625 mg/day) was given for the first 4 weeks; this was increased to the target dose of Bosentan and was well tolerated. Bosentan was also used in two neonates with d transposition of great arteries and was found to be safe and effective.[5]

Our experience with Bosentan in the post-operative situation in this patient was favorable. As there are no specific guidelines for the dosage of Bosentan in neonates, we used the dose of 15.625 mg/day,[4] which was well tolerated with no appreciable side effects. The efficacy cannot be proven with certainty but the striking drop in PA pressure and the absence of PAH crisis correlated with the anticipated peak of Bosentan action. iNO could be weaned off successfully only after starting Bosentan.

The side effects are a dose-dependent asymptomatic increase in liver enzymes, mild anemia, and pedal edema.[3,4] A disadvantage of Bosentan is its prohibitive cost and difficulty in procurement.

Further studies are required to determine the dosage and document efficacy of Bosentan in the post-operative neonate with PAH.
